# Chain of Call: Learning How to Effectively Communicate with Emergency Medical Services at School

**DOI:** 10.3390/children12111501

**Published:** 2025-11-05

**Authors:** Santiago Martínez-Isasi, Cristina Jorge-Soto, Cristina Varela-Casal, María Fernández-Méndez, María García-Martínez, Adriana Seijas-Vijande, Carlos Berlanga-Macías, María Pichel-López, Carmen Agra-Tuñas, Antonio Rodríguez-Núñez

**Affiliations:** 1 CLINURSID Research Group, Radiology, Psychiatry, Public Health, Nursing and Medicine Department, Universidade de Santiago de Compostela, 15705 Santiago de Compostela, Spain; santiago.martinez.isasi@usc.es (S.M.-I.);; 2 (SICRUS) Research Group, Health Research Institute of Santiago, University Hospital of Santiago de Compostela-CHUS, 15706 Santiago de Compostela, Spain; 3 Primary Care Interventions to Prevent Maternal and Child Chronic Diseases of Perinatal and Developmental Origin (RICORS), Instituto de Salud Carlos III, RD21/0012/0025, 28029 Madrid, Spain; 4 Faculty of Nursing, University of Santiago de Compostela, 15782 Santiago de Compostela, Spain; 5 REMOSS Research Group, Faculty of Education and Sport Sciences, University of Vigo, 36310 Vigo, Spain; cristinavarelacasal@uvigo.es (C.V.-C.);; 6 Faculty of Design, University of Vigo, 36310 Vigo, Spain; 7 School of Nursing of Pontevedra, University of Vigo, 36001 Pontevedra, Spain; 8 Health and Social Research Center, Universidad de Castilla-La Mancha, 16002 Cuenca, Spain; 9 Faculty of Nursing, Universidad de Castilla-La Mancha, 02001 Albacete, Spain; 10 Complexo Hospitalario Universitario de A Coruña (CHUAC), Sergas, Universidade da Coruña (UDC), 15006 A Coruña, Spain; 11 Complexo Hospitalario Universitario de Santiago de Compostela (CHUS), 15706 Santiago de Compostela, Spain

**Keywords:** basic life support, schoolchildren, training, simulation, cardiopulmonary resuscitation

## Abstract

**Highlights:**

**What are the main findings?**

- The use of didactic materials specifically developed by the research team is effective in helping schoolchildren acquire emergency call skills.
- A sequential learning approach allows the training to be tailored to the cognitive development and learning capacity of different age groups.

**What is the implication of the main finding?**

- This methodology enables schoolchildren to recognise emergency situations and initiate an EMS call, although some challenges remain in activating the hands-free function.
- These findings support the development of evidence-based recommendations for basic life support training in school settings.

**Abstract:**

**Background/Objectives**: More than half of out-of-hospital cardiac arrests occur at home and are witnessed by family members, who must promptly call the Emergency Medical Services (EMS). The aim of this study was to assess the learning outcomes of an interactive school-based training activity focused specifically on the EMS call. **Methods**: A single-group, post-test-only simulation study was conducted in five Spanish schools. Participating schoolchildren received basic life support (BLS) training from their Physical Education teachers, integrated into the regular school schedule and following the Kids Save Lives recommendations. An innovative didactic resource (the “BLS Endless Book”) was used to support active learning. Children’s performance was evaluated in a simulated scenario using a standardized checklist. **Results**: A total of 1341 children aged 6 to 14 years participated. In the simulated scenario, more than 90% of participants were able to recognize the emergency and correctly identified and dialed the national emergency number. However, less than 50% were able to activate the hands-free function (with younger children experiencing more difficulty). During the call, 99.0% stated their full name, and 82.0% provided their complete address. **Conclusions**: A short, focused BLS training led at school by physical education teachers and based on an interactive, easy-to-use, didactic tool is effective in educating 6–14-year-old schoolchildren to correctly perform an immediate EMS call in case of cardiac arrest. Nevertheless, schoolchildren may require reinforcement training focused on hands-free operation and on providing the correct address.

## 1. Introduction

More than half of all cardiac arrests occur in out-of-hospital settings, most commonly at home and in the presence of relatives. While bystander cardiopulmonary resuscitation (CPR) rates for out-of-hospital cardiac arrests (OHCAs) have increased significantly over the past two decades, the key determinant of survival is the timeliness of intervention, rather than the identity of the responder. Given that the majority of OHCAs take place in private residences, there is a critical need to expand basic life support (BLS) training among the general public to improve outcomes. Rapid return of spontaneous circulation (ROSC) is critical for survival regardless of the type of rescuer, although CPR initiated by bystanders generally takes longer (20 min vs. 5 min for EMS) and more often requires advanced airway management. Therefore, the immediacy of the response is essential to improve survival rates and prevent long-term sequelae [1]. To address this, the scientific community supports first aid education for the general population, identifying children and schools as ideal targets for this training [1,2,3]. In 2015, the Kids Save Lives statement from the European Resuscitation Council (ERC) (endorsed by the World Health Organization (WHO) and other scientific bodies) outlined the key principles of BLS instruction in schools [4,5]. Since then, BLS education has been promoted and gradually implemented in schools across various countries and regions [5,6,7,8].

The first and relatively larger link in the chain of survival is “early recognition and call for help” [3,5,9]. Consequently, laypersons should be able to immediately and effectively call and communicate with Emergency Medical Services (EMS) in the event of cardiac arrest. In the case of schoolchildren, based on limited evidence and expert consensus [5], it has been suggested that children as young as 4 years old should be taught the emergency number [3,10,11]. However, it is surprising that teaching how to properly alert EMS has not been systematically included in BLS school training programs [10,12,13]. Thus, the present study aimed to evaluate the learning outcomes of an innovative and interactive teaching methodology focused on emergency calls among schoolchildren aged 6 to 14 years.

## 2. Materials and Methods

### 2.1. Design

**Figure 1:**
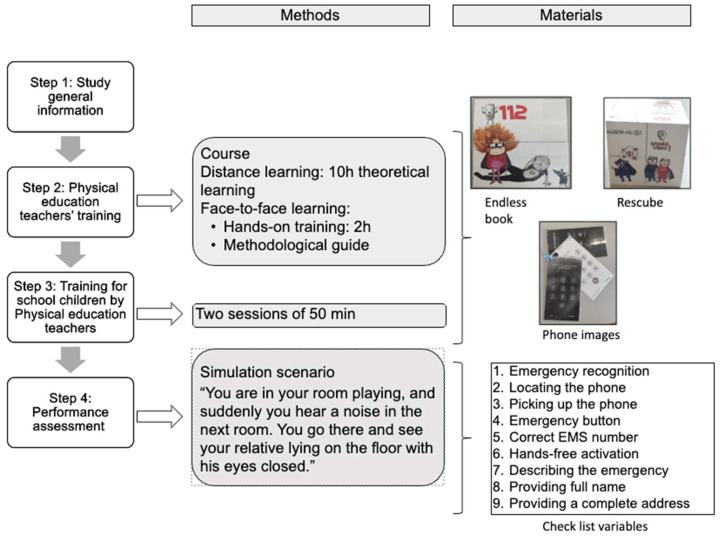
Study design, materials, and assessment.

A single-group, post-test-only simulation study was conducted and carried out in four steps (Figure 1).

### 2.2. Participants

This study involved 1483 children aged 6 to 14 years (primary education: 6–12 years old; secondary education: 13–14 years old) attending five public charter schools in Galicia (Spain) during the 2021–2022 academic year. 

Children with any physical or mental condition that could affect their understanding or performance of the skills received the training but were excluded from the analysis.

Schools were selected through convenience sampling based on the number of pupils and their availability. Recruitment began with a request for participation addressed to each school’s head teacher, who granted permission to conduct the study. Following approval, an invitation letter was sent to the legal guardians of all children aged 6 to 14 years. Participation required written informed consent from the guardians and verbal assent from the children. 

The research protocol was approved by the Ethics Committee of the Faculty of Education and Sport Sciences—University of Vigo (Spain) (Code: 09-170123).

### 2.3. Intervention

The design of the first two steps is standard to our previously published study on foreign body airway obstruction training [14], and was developed in accordance with ERC guidelines [15]. A brief description is provided below:

**Steps 1 (general study information) and 2 (training of physical education teachers) were carried out as described in the article of Martinez-Isasi et al. [14].** The first step consisted of holding meetings with the management teams of the five participating schools and the children’s legal guardians to provide information about the study. In the second step, physical education teachers received hybrid training (10 h of distance learning and 2 h of face-to-face hands-on training) delivered by three BLS instructors from the research team.

**Step 3: Training of schoolchildren by PE teachers.** The training content was standardised through a guide that outlined the common content, methodology, structure, and timing of the sessions. The didactic approach followed the sequence: explain–demonstrate–practice. Training duration ranged from two to four hours, depending on the grade and content to be taught. For this purpose, training was based on the learning model proposed by De Buck [10], in which competencies are progressively added according to the developmental stage. Thus, the training programme begins in 1st grade with emergency recognition and calling EMS. CPR is then introduced in 3rd grade, followed by the recovery position in 5th grade and training on foreign body airway obstruction (FBAO) in 6th grade. From secondary school onwards, defibrillator use is also incorporated.

Therefore, each academic year builds on the competencies developed in previous years, creating a sequential learning process. 

Emergency call training was carried out in two 50-min sessions in 1st grade of elementary education and became part of the training curriculum in subsequent grades. The first session focused on knowledge acquisition. To reinforce active learning, students used the didactic resource Endless Book, developed from Rescube, a previously validated tool created by the research group for school-based BLS training [16]. The second session involved four brief simulation scenarios focused on EMS calling and communication in case of cardiac arrest, conducted in groups of 25 students (primary) and 30 students (secondary) per teacher.

The 112 call training included a presentation of phone images and the use of a teacher’s phone. The simulated call consisted of three steps: unlocking the phone, calling 112, and activating the hands-free function.

**Step 4: Performance assessment.**

Students were assessed using an adult cardiac arrest simulation scenario, which took place between 7 and 15 days after training. No pre-training assessment was conducted; the post-training evaluation was the only one performed in this study. The child was told: “*You are in your room playing, and suddenly you hear a noise in the next room. You go there and see your relative lying on the floor with his eyes closed.*” Next to an adult BLS manikin, there was a mock-up of a mobile phone with a locked screen. When the child checked the victim’s consciousness, the instructor indicated that the victim was unresponsive and not breathing. The case ended when the child provided the location to the EMS dispatcher (role played by the instructor). As an assessment tool, a predefined checklist including the trained steps was used (Figure 1). Each assessment session was conducted by a research team composed of a general coordinator and six trained evaluators (18 in total) [14].

The schoolchildren used a low-cost telephone to call the emergency medical system (112). This phone required two steps.

### 2.4. Teaching and Training Materials

In this study, an adapted teaching tool (Call the EMS Endless Book) was used for training [16], along with a low-cost, paper-based mobile phone mock-up for skills assessment.

### 2.5. Assessment Checklist (Figure 1)

The following items were recorded as present/absent [10]:

- Emergency recognition: recognising the case as an emergency where it is necessary to alert emergency services.
- Locating the phone: locating the phone at the scene.
- Picking up the phone: deciding to call the emergency service.
- Emergency button: pressing the emergency call button on the screen of a locked mobile phone.
- Correct EMS number: dialling the correct emergency number (112).
- Hands-free activation: pressing the corresponding icon on the phone model to activate hands-free mode.
- Describing the emergency.
- Providing full name.
- Providing a complete address.

### 2.6. Statistical Analysis

All analyses were performed with IBM SPSS Statistics version 25.0 for macOS. Results were expressed as absolute frequencies (relative frequencies) as appropriate. The clustering variables were “academic grade” and “educational level”. In the first case, each grade was considered a category of the variable. The variable “educational level” is a dichotomous qualitative variable categorised as “elementary education” (EE) and “secondary school” (SS), where grades 1 to 6 of elementary education comprise the first category and grades 1 and 2 of secondary school comprise the second category. In cases where a much lower level of performance was observed in younger schoolchildren (1st grade of elementary education), those data were included in the comparison by grade but were not included in the ‘elementary education’ group.

Hypothesis testing was performed using chi-square and Fisher’s exact tests to compare proportions between groups. For all analyses, *p* < 0.05 was considered statistically significant. However, in post hoc pairwise comparisons, the Bonferroni correction was applied to control for Type I error inflation due to multiple testing. In these cases, the adjusted significance threshold was set at α = 0.05/m (where m is the total number of comparisons), requiring *p* < 0.00179 for statistical significance. For all comparisons, relative risk (RR), effect sizes (ES), and the 95% confidence interval were calculated. Effect size was interpreted using Cramer’s V: (0.10–0.30) small effect size; (0.30–0.50) moderate effect size; (≥0.50) large effect size. Logistic regression was used for the multivariate analysis.

## 3. Results

Of the 1483 schoolchildren trained and invited to participate in this study, 142 were excluded for missing or incorrect data, and the data from 1341 (52.3% girls) were analysed. Participants belonged to two educational levels: elementary education (EE) (ages 6 to 11) and secondary school (SS) (ages 12 to 14). All grades from EE (grades 1 to 6) and the first two grades of SS (grades 1 and 2) were included in the study. The distribution of the sample by school grade is shown in Table 1.

**Figure 2:**
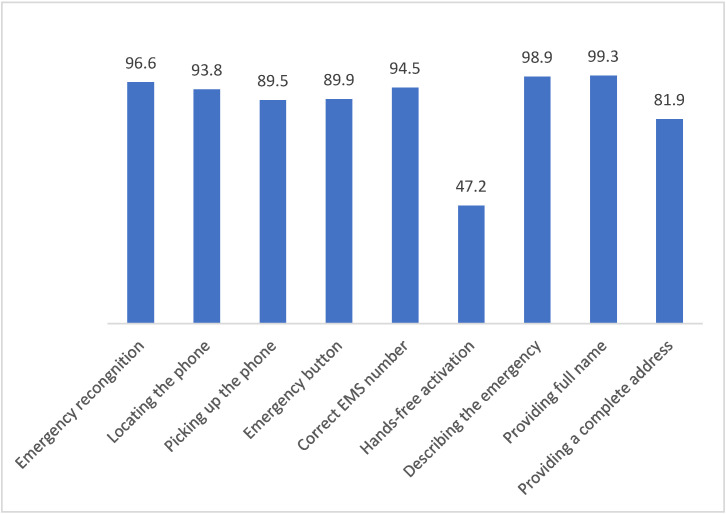
Correct performance in sample. Data expressed as percentages.

The overall performance (Figure 2) shows that all the skills were achieved by more than 80% of the sample, except for the hands-free variable, which was achieved by less than 50% of participants, entailing the most common failure.

**Figure 3:**
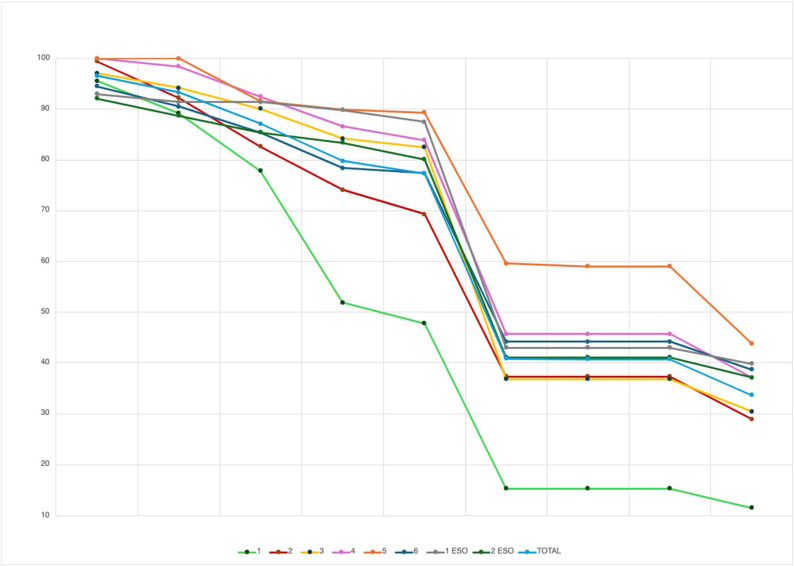
Graphical representation of participants who completed the sequence correctly.

To analyse the performance of each variable by educational level (Elementary Education vs. Secondary School), the data from the 158 youngest participants were excluded, as their performance differed significantly from that of the other groups, as shown in Figure 3.

Thus, when comparing the two educational levels (Table 2) significant differences were found in four variables: emergency recognition (EE 98.1% vs. SS 92.5%, *p* < 0.001), locating the phone (EE 95.5% vs. SS 90.7%, *p* = 0.003), providing full name (EE 99.7% vs. SS 98.6%, *p* = 0.036) and providing complete address (EE 82.8% vs. SS 91.8%, *p* < 0.001). However, the analysis of RR and effect size did not indicate any impact of educational level on skill performance.

Nevertheless, despite the statistical differences, all of these skills were correctly performed by more than 90% of participants in each group, except for providing a complete address, where 82% of EE participants gave full and correct information. No significant differences were found in hands-free activation; this skill was correctly performed by around 50% of both EE and SS participants, making it the most common error. 

Binomial logistic regression analysis showed that 5 of the 9 variables were statistically significant (*p* < 0.05) and contributed to predicting emergency call skills (Table 3).

Figure 3 and Table S1 (see Supplementary Materials) show the percentage of participants in each academic grade who correctly completed the full sequence (from emergency recognition to providing a full address during the EMS call). 

Each line represents the progress of a specific academic grade, showing the percentage of participants who failed each step. Therefore, the endpoint of the line represents the proportion of schoolchildren who completed the entire sequence without failure. 

Thus, the percentage of schoolchildren who successfully complete the entire emergency call sequence is around 40% in secondary school and in the upper years of elementary education (grades 4, 5, and 6) and around 30% in grades 2 and 3 of elementary education. The lowest percentage (11.5%) is found among 1st-grade students. The exact percentages for each step are provided in the.

Significant differences were found when comparing each academic grade with the others across the assessed items using the Chi-square test. The specific values are shown in Table S2 (see Supplementary Materials). However, the analysis of RR and effect size revealed significant differences in the following comparisons: “Emergency recognition” (4th vs. 1st SE; 4th vs. 2nd SE; 5th vs. 1st SE; 5th vs. 2nd SE), “Locating the phone” (5th vs. 1st SE; 5th vs. 2nd SE), “Emergency button” (1st vs. 5th), and “Describing the emergency” (4th vs. 2nd SE).

## 4. Discussion

Kid Save Lives is a global, multifaceted initiative aimed at improving OHCA outcomes by training schoolchildren, considering them the future informed, aware, and skilled bystanders [3,4,5,6,7,8,17,18,19]. Our results show that younger, first-time learners (1st grade of elementary education) make more mistakes than other schoolchildren. Additionally, the data highlight two critical weaknesses: hands-free activation, where nearly half of participants across all grades struggle, and providing a full address, where performance drops more noticeably in elementary grades compared to secondary school.

Our results are consistent with the limited existing studies, which show that a child should be able to technically use a phone, know how to describe their location, and synthesise a situation by the age of 9–10 years [10,20]. Although young children are increasingly exposed to multiple screens, including smartphones and tablets, the rapid spread of mobile screen devices in recent years may explain a common mistake observed across all academic grades: failing to activate the hands-free function [21].

Previous studies on BLS training in schoolchildren have shown similar results [22]. Bolling et al. [23] found that after training, 77% of schoolchildren aged 6–7 years knew the correct EMS number. In contrast, their training program consisted of five 45-min lessons, significantly more than in our study, where schoolchildren received only two 50-min sessions.

Even though young children are familiar with mobile devices, Hubber et al. [11] reported that, in a simulated situation, schoolchildren aged 4–6 years could not complete the emergency call and report the emergency to the dispatcher. However, half of them were able to recognise the emergency situation. Similarly, although schoolchildren aged 7–9 years mostly identified the emergency, only 16% completed the emergency report. It should be noted that participants were not trained before the simulation test, unlike in our study, which shows that a brief and simple training with specific didactic material is useful for improving schoolchildren’s skills.

Therefore, the fact that children are familiar with the playful use of mobile devices should not be confused with having the skills to make an emergency call correctly.

The 2015 ERC Resuscitation Guidelines [24] included, for the first time, the recommendation to activate hands-free mode during the emergency call. This was based on the idea that doing so would make it easier for the rescuer to follow the dispatcher’s instructions, thereby facilitating the initiation of CPR. To date, however, no previous study has analysed the ability to activate hands-free mode in the general population or among schoolchildren. In our study, we observed that this step represents a barrier to the correct execution of the life support sequence, as half of the participants failed at this stage, regardless of age. Therefore, based on our results, we consider it important that future resuscitation guidelines reinforce this aspect, recommending that specific time be devoted to hands-free training in school programs and that it be reviewed during retraining sessions. 

When comparing emergency call skills across academic levels, we observed that, overall, the only significant difference between elementary education and secondary school is in providing a full address. In this case, younger schoolchildren showed a lower level of performance, with this being the second most common error in this group, as it was the only skill (apart from activating the hands-free function) that was correctly executed by less than 90% of participants.

Our study, involving around 1400 schoolchildren aged 6 to 14 years, is the first to analyse emergency call skills in a large cohort of schoolchildren over a wide age range the after a teaching program conducted by physical education teachers at schools, using simple interactive methods. Our main result indicates that after a brief explanation and simulation training, the great majority of children (regardless of their age) successfully completed an effective call to EMS during the simulation challenge. Our findings are consistent with the available evidence, which supports the idea that schoolchildren benefit from teaching materials adapted to their level of psychological development [3,5,14,16].

Despite these positive results, we also identified some weak points in the chain-of-calling that require reinforcement training, especially in the youngest children: they found difficulties activating hands-free mode and providing their complete address. These gaps may be critical in real cases, considering that if a link breaks, the chain-of-calling becomes ineffective [25]. Thus, schoolchildren may require reinforcement training focused on hands-free operation and on providing the correct address.

This model is based on certified instructors training physical education teachers, who have been identified by the ERC as the most suitable trainers for schoolchildren. Training teachers instead of relying on certified healthcare professionals facilitates implementation and addresses one of the main challenges in bringing BLS training into schools.

As future lines of research, we consider it important to follow up with this cohort over time. This line of research will allow us to assess knowledge retention and the effectiveness of retraining, as well as identify areas for improvement in skills where the most errors were observed.

### Limitations

This study was carried out in a specific geographical and cultural area in public charter schools, which hampers the generalizability of the results. Furthermore, the absence of a control group and the reliance on descriptive statistics limit the ability to support causal inference. Also, the chosen CA simulated scenario, although realistic, does not reproduce a real-life situation where layperson stress and environmental conditions could affect children’s performance. Additionally, we did not analyse whether the schoolchildren had prior knowledge of life support, although life support training was not previously established in the participating schools. Therefore, if any student had any knowledge, it would not have come from formal sources, so we consider that this aspect would not affect the interpretation of our results. In addition, knowledge and skill retention over time was not assessed, nor was any comparison of performance levels before and after training, as the practical assessment was only carried out between 7 and 15 days after receiving the training.

## 5. Conclusions

A short, focused BLS training session led by physical education teachers at school and based on an interactive, easy-to-use, didactic tool is effective in educating 6–14-year-old schoolchildren to correctly perform an immediate EMS call in case of cardiac arrest. Nevertheless, schoolchildren may require reinforcement training focused on hands-free operation and on providing the correct address. Therefore, it could be interesting to include this methodology in the school curriculum and periodically assess its strengths and impact on public health.

## Figures and Tables

**Table 1:** Sample distribution by school grade. Data are expressed as absolute frequencies and percentages.

Academic Grade	N	%
1st EE ^A^	158	11.8
2nd EE	167	12.5
3rd EE	173	12.9
4th EE	186	13.9
5th EE	178	13.3
6th EE	199	14.9
1st SS ^B^	128	9.6
2nd SS	151	11.3

^A^ Elementary Education. ^B^ Secondary School.

**Table 2:** Comparison of each variable by educational level. Data expressed as frequency (percentage).

Variable	Elementary Education (N = 903)	Secondary School (N = 279)	*p* Value ^a^	RR ^b^ (95% CI)	Effect Size ^c^
Emergency recognition	886	258	<0.001	2.45	0.136
	(98.1)	(92.5)		(1.80; 3.32)	
Locating the phone	862	253	0.003	1.71	0.088
	(95.5)	(90.7)		(1.24; 2.35)	
Picking up the phone	815	253	0.91	-	-
	(90.5)	(90.7)			
Emergency button	838	260	0.871	-	-
	(92.9)	(93.2)			
Correct EMS number	867	264	0.318	-	-
	(96)	(94.6)			
Hands-free activation	455	129	0.225	-	-
	(50.4)	(46.2)			
Describing the emergency	897	274	0.086	-	-
	(99.3)	(98.2)			
Providing full name	900	275	0.036	2.44	0.061
	(99.7)	(98.6)		(1.27; 4.67)	
Providing complete address	748	256	<0.001	1.16	0.106
	(82.8)	(91.8)		(1.09; 1.25)	

^a^ Chi-Square test; ^b^ RR for the lowest-performing group; ^c^ Cramer’s V.

**Table 3:** Binomial logistic regression.

	Gender		Grade		Model	
	OR (IC95%)	*p* Value	OR (IC95%)	*p* Value	*p* Value	R ^a^
Emergency recognition	1.196 (0.621–2.302)	0.591	0.233 (0.121–0.449)	0.000	0.000	0.063
Locating the phone	1.882 (1.132–3.126)	0.015	0.445 (0.266–0.744)	0.002	0.001	0.034
Picking up the phone	1.810 (1.215–2.694)	0.004	0.995 (0.627–1.580)	0.982	0.013	0.016
Emergency button	2.051 (1.290–3.266)	0.002	1.006 (0.590–1.714)	0.983	0.008	0.020
Correct EMS number	1.638 (0.926–2.899)	0.090	0.711 (0.383–1.321)	0.281	0.144	0.011
Hands-free activation	1.238 (0.984–1.556)	0.068	0.837 (0.639–1.097)	0.195	0.090	0.005
Describing the emergency	3.145 (0.828–11.95)	0.093	0.345 (0.104–1.142)	0.082	0.057	0.048
Providing Full name	3.015 (0.580–15.68)	0.190	0.216 (0.048–0.975)	0.046	0.062	0.067
Providing Complete address	1.159 (0.840–1.598	0.368	2.289 (1.444–3.628)	0.000	0.000	0.023

^a^ binary logistic regression model.

## Data Availability

The original contributions presented in this study are included in the article/Supplementary Materials. Further inquiries can be directed to the corresponding author.

## Supplementary Materials

The following supporting information can be downloaded at: https://www.mdpi.com/article/10.3390/children12111501/s1, Table S1: Percentage of participants in each academic grade with correct performance; Table S2: intragroup analysis academic grade comparison on each item assessed. Data expressed in absolute frequency and percentage.

## Abbreviations

The following abbreviations are used in this manuscript:

EMS	Emergency medical system
BLS	Basic life support
EE	Elementary education
SS	Secondary school
