# Supplementary material for: Chain of Call: Learning How to Effectively Communicate with Emergency Medical Services at School
Source: Children (Basel). 2025 Nov 5;12(11):1501. doi: 10.3390/children12111501 (PMC12651430; doi:10.3390/children12111501)
Supplement: Supplementary file 1 [file children-12-01501-s001.zip › children-3930779-supplementary.pdf]

children

MDPI

Article

Chain-of call: Learning at school how to effectively communicate with Emergency Medical Services.

Supplementary material S1

Table S1: Percentage of participants in each academic grade with correct performance.

	1st EE	2nd EE	3th EE	4th EE	5th EE	6th EE	1st SS	2nd SS	Total
Step 1	95,6	99,4	97,1	100	100	94,5	93	92,1	96,6
Steps 1-2	89,2	92,2	94,2	98,4	100	90,5	91,4	88,7	93,3
Steps 1-3	77,8	82,6	90,1	92,5	91,6	85,4	91,4	85,4	87,1
Steps 1-4	51,9	74,1	84,2	86,6	89,9	78,4	89,8	83,4	79,8
Steps 1-5	47,8	69,3	82,5	83,9	89,3	77,4	87,5	80,1	77,3
Steps 1-6	15,3	37,3	36,8	45,7	59,6	44,2	43	41,1	40,8
Steps 1-7	15,3	37,3	36,8	45,7	59	44,2	43	41,1	40,7
Steps 1-8	15,3	37,3	36,8	45,7	59	44,2	43	41,1	40,7
Steps 1-9 (Full sequence)	11,5	28,9	30,4	37,1	43,8	38,7	39,8	37,1	33,6

Step 1: Emergency recognition. Step 2: Phone location. Step 3: Picking up the phone. Step 4: Emergency button. Step 5: correct EMS number. Step 6: Hands-free activation.

Step 7: Describing the emergency. Step 8: Providing full name. Step 9: Providing complete address.

Supplementary material S2

Table S2: intragroup analysis academic grade comparison on each item assessed. Data expressed in absolute frequency and percentage.

Variable	Grades	Correct performance, p value[a]	RR (95% CI)[b]	Effect size[c]
Emergency recognition	2nd vs 2nd SE	166 (99.4) vs. 139 (92.1) p<0.001	2.02 (1.66; 2.47)	0.185
	4th vs 1st SE	186 (100) vs 119 (93) p<0.001	2.56 (2.22; 2.94)	0.207
	4th vs 2nd SE	186 (100) vs 139 (92,1) p<0.001	2.33 (2.06; 2.65)	0.213
	5th vs 6th	178 (100) vs 188 (94.5) p<0.001	1.94 (1.76; 2.15)	0.164
	5th vs 1st SE	178 (100) vs 119 (93) p<0.001	2.49 (2.7; 2.86)	0.205
	5th vs 2nd SE	178 (100) vs 139 (92,1) p<0.001	2.28 (2.01; 2.58)	0.211
Locating phone	1st vs 4th	142 (89.9) vs. 183 (98.4) p<0.001	1.92 (1.53; 2.42)	0.186
	1st vs 5th	142 (89.9) vs. 178 (100) p<0.001	2.24 (1.99; 2.54)	0.237
	2nd vs 5th	154 (92.2) vs. 178 (100) p<0.001	2.15 (1.92; 2.42)	0.204
	4th vs 6th	183 (98,4) vs 181 (91) p<0,001	1.72 (1.40; 2.11)	0.164
	4th vs 2nd SE	183 (98,4) vs 135 (89,4) p<0,001	1.98 (1.57; 2.50)	0.194
	5th vs 6th	178 (100) vs 181 (91) p<0,001	1.98 (1.79; 2.19)	0.212
	5th vs 1st SE	178 (100) vs 118 (92,2) p<0,001	2.50 (2.8; 2.88)	0.217
	5th vs 2nd SE	178 (100) vs 135 (89,4) p<0,001	2.31 (2.04; 2.63)	0.245
Emergency button	1st vs 2nd	106 (67.1) vs 145 (87.3) p<0.001	1.68 (1.37; 2.07)	0.242
	1st vs 3th	106 (67.1) vs. 161 (93.1) p<0.001	2.04 (1.69; 2.47)	0.329
	1st vs 4th	106 (67.1) vs. 175 (94.1) p<0.001	2.18 (1.81; 2.64)	0.348
	1st vs 5th	106 (67.1) vs. 175 (98.3) p<0.001	2.50 (2.12; 2.95)	0.421

	1st vs 6th	106 (67.1) vs. 182 (91.5) p<0.001	2.04 (1.67; 2.50)	0.307
	1st vs 1st SE	106 (67.1) vs. 122 (95.3) p<0.001	1.92 (1.63; 2.27)	0.349
	1st vs 2nd SE	106 (67.1) vs. 138 (91.4) p<0.001	1.84 (1.52; 2.22)	0.298
	2nd vs 5th	145 (87.3) vs. 175 (98.3) p<0.001	1.93 (1.59; 2.34)	0.215
**Correct EMS number**	1st vs 3th	135 (86) vs. 166 (96) p<0.001	1.69 (1.33; 2.15)	0.176
	1st vs 4th	135 (86) vs. 181 (97.3) p<0.001	1.90 (1.53; 2.37)	0.210
	1st vs 5th	135 (86) vs. 177 (99.4) p<0.001	2.21 (1.89; 2.57)	0.265
	1st vs 6th	135 (86) vs. 161 (96) p<0.001	1.77 (1.37; 2.27)	0.179
	2nd vs 5th	152 (91) vs. 177 (99.4) p<0.001	2.02 (1.70; 2.40)	0.200
**Hands-free activation**				
	1st vs 4th	49 (31) vs. 95 (51.1) p<0.001	1.60 (1.23; 2.07)	0.203
	1st vs 5th	49 (31) vs. 112 (62.9) p <0.001	2.04 (1.57; 2.65)	0.319
	1st vs 6th	49 (31) vs. 99 (49.7) p<0.001	1.57 (1.21; 2.05)	0.189
	3th vs 5th	69 (39,9) vs 112 (62,9) p<0.001	1.60 (1.28; 2.00)	0.230
**Providing complete address**	1st vs 3th	93 (58.9) vs. 137 (79.2) p<0.001	1.59 (1.28; 1.97)	0.221
	1st vs 4th	93 (58.9) vs. 157 (84.4) p<0.001	1.85 (1.50; 2.29)	0.286
	1st vs 5th	93 (58.9) vs. 142 (79.8) p<0.001	1.62 (1.31; 2.01)	0.228
	1st vs 6th	93 (58.9) vs. 180 (90.5) p<0.001	2.27 (1.85; 2.77)	0.370
	1st vs 1st SE	93 (58.9) vs. 118 (92.2) p<0.001	1.96 (1.64; 2.34)	0.377
	1st vs 2nd SE	93 (58.9) vs. 138 (91.4) p<0.001	2.07 (1.71; 2.49)	0,374

[a]Chi Square test, significance level adjusted by Bonferroni test p<0,0018; [b]RR for the worst performing group;
[c]Cramer´s V

Supplementary material S3

TREND Statement checklist

Paper Section/ Topic	Item No	Descriptor	Reported?	
			✓	Pg #
**Title and Abstract**			x	1
Title and Abstract	1	• Information on how units were allocated to interventions	x<br>x	1-2<br>1
		• Structured abstract recommended	x	2
		• Information on target population or study sample	x	2
**Introduction**			x	2-3
Background	2	• Scientific background and explanation of rationale	x	2
		• Theories used in designing behavioral interventions	x<br>x	2<br>2
**Methods**				
Participants	3	• Eligibility criteria for participants, including criteria at different levels in recruitment/sampling plan (e.g., cities, clinics, subjects)	x<br>x<br>x<br>x	3-4<br>3-4<br>3-4<br>3-4

3-4

3-4

		• Method of recruitment (e.g., referral, self-selection), including the sampling method if a systematic sampling plan was implemented		
		• Recruitment setting		
		• Settings and locations where the data were collected		
Interventions	4	• Details of the interventions intended for each study condition and how and when they were actually administered, specifically including:		
		○ Content: what was given?		
		○ Delivery method: how was the content given?		
		○ Unit of delivery: how were the subjects grouped during delivery?		
		○ Deliverer: who delivered the intervention?		
		○ Setting: where was the intervention delivered?		
		○ Exposure quantity and duration: how many sessions or episodes or events were intended to be delivered? How long were they intended to last?	x x	
		○ Time span: how long was it intended to take to deliver the intervention to each unit?	x	3-4
		○ Activities to increase compliance or adherence (e.g., incentives)		
Objectives	5	• Specific objectives and hypotheses	x x	2 4-5
Outcomes	6	• Clearly defined primary and secondary outcome measures		4-5 x
		• Methods used to collect data and any methods used to enhance the quality of measurements		

		• Information on validated instruments such as psychometric and biometric properties		
Sample Size	7	• How sample size was determined and, when applicable, explanation of any interim analyses and stopping rules		
Assignment Method	8	• Unit of assignment (the unit being assigned to study condition, e.g., individual, group, community)		
		• Method used to assign units to study conditions, including details of any restriction (e.g., blocking, stratification, minimization)		
		• Inclusion of aspects employed to help minimize potential bias induced due to non-randomization (e.g., matching)		
